# Traumatic Hip Dislocations in Major Trauma Patients: Epidemiology, Injury Mechanisms, and Concomitant Injuries

**DOI:** 10.3390/jcm11030472

**Published:** 2022-01-18

**Authors:** Christian D. Weber, Rolf Lefering, Richard M. Sellei, Klemens Horst, Filippo Migliorini, Frank Hildebrand, TraumaRegister DGU

**Affiliations:** 1 Department of Orthopaedics, Trauma and Reconstructive Surgery, RWTH Aachen University, 52074 Aachen, Germany; khorst@ukaachen.de (K.H.); migliorini.md@gmail.com (F.M.); fhildebrand@ukaachen.de (F.H.); 2 Institute for Research in Operative Medicine, Witten/Herdecke University, 58448 Witten, Germany; rolf.lefering@uni-wh.de; 3 Department of Trauma and Orthopaedic Surgery, Sana Clinic, 63069 Offenbach, Germany; richard.sellei@sana.de

**Keywords:** traumatic hip dislocation, orthopedic emergency, injury mechanisms, acetabular fracture, aortic injury, ischial nerve injury

## Abstract

Introduction: Traumatic hip dislocations (THDs) are severe injuries associated with considerable morbidity. Delayed recognition of fracture dislocations and neurovascular deficits have been proposed to cause deleterious long-term clinical outcomes. Therefore, in this study, we aimed to identify characteristics of epidemiology, injury mechanisms, and associated injuries to identify patients at risk. Methods: For this study based on the TraumaRegister DGU^®^ (January 2002–December 2017), the inclusion criterion was an Injury Severity Score (ISS) ≥9 points. Exclusion criteria were an isolated head injury and early transfer to another hospital. The THD group was compared to a control group without hip dislocation. The ISS and New ISS were used for injury severity and the Abbreviated Injury Scale for associated injuries classification. Univariate and logistic regression analyses were performed. Results: The final study cohort comprised *n* = 170,934 major trauma patients. We identified 1359 individuals (0.8%) with THD; 12 patients had sustained bilateral hip dislocations. Patients with THD were predominantly male (79.5%, mean age 43 years, mean ISS 22.4 points). Aortic injuries (2.1% vs. 0.9%, *p* ≤ 0.001) were observed more frequently in the THD group. Among the predictors for THDs were specific injury mechanisms, including motor vehicle accidents (odds ratio (OR) 2.98, 95% confidence interval (CI) 2.57–3.45, *p* ≤ 0.001), motorcycle accidents (OR 1.99, 95% CI 1.66–2.39, *p* ≤ 0.001), and suicide attempts (OR 1.36, 95% CI 1.06–1.75, *p* = 0.016). Despite a lower rate of head injuries and a comparable level of care measured by trauma center admission, both intensive care unit and total hospital stay were prolonged in patients with THD. Conclusions: Since early diagnosis, as well as timely and sufficient treatment, of THDs are of high relevance for long-term outcomes of severely injured individuals, knowledge of patients at risk for this injury pattern is of utmost importance. THDs are frequently related to high-energy mechanisms and associated with severe concomitant injuries in major trauma patients.

## 1. Introduction

Traumatic hip dislocations (THDs) represent a significant orthopedic emergency [1,2,3]. They are subdivided into “simple” and “complex” dislocations, depending on the presence of concomitant skeletal and/or soft-tissue injuries. The nature of concomitant injuries and the time interval until achieving anatomic reduction both affect the complication rate and long-term functional outcomes [4]. Therefore, prompt reduction and immediate recognition of associated injuries are considered to be fundamental principles in the management of this injury pattern to avoid morbidity and malpractice allegations [5].

The potentially devastating long-term effects of THD have been investigated in different studies. Pape et al. evaluated multiply injured patients with THDs in the 1990s and observed the development of avascular necrosis even in “simple” dislocations [5]. Various long-term sequelae have been characterized in the literature, including post-traumatic osteoarthritis of the hip, avascular necrosis of the femoral head, periarticular ossifications, recurrent dislocation, and ischial nerve palsy [6,7,8,9,10,11].

Previous studies that have focused on THDs in the acute post-traumatic setting are sparse and based on rather small sample sizes. Two decades ago, Hak et al. observed, in a study cohort of 66 patients, that most THDs particularly affected unrestrained drivers or passengers of motor vehicles [12]. The incidence of associated injuries was reported to be as high as 95%, and additional orthopedic injuries were observed in 33% of patients. More recent studies have suggested that this injury pattern is still a major issue in victims of road traffic accidents, despite recent road safety initiatives and modern restraint devices [13], or even indicated increased incidence rates in North America [14]. In 1990, Marymont et al. characterized a previously unrecognized injury complex of posterior hip dislocations and acute traumatic injuries of the thoracic aorta [15]. A group from Houston reported a series of 89 patients with posterior hip dislocation, including 7 individuals (8%) with an aortic injury after deceleration trauma. All patients presented hemodynamically stable during the initial evaluation. More recent studies have also reported on combined hip dislocations and aortic injuries but have been unable to perform statistical analysis due to the rarity of this life-threatening injury pattern [12,13].

However, recent data about the epidemiology, injury mechanisms, and associated injury based on a large study cohort for the identification of patients at risk are unavailable. Furthermore, the rate of delayed diagnosis and reduced THD, as well as the impact of THD on the acute posttraumatic course, remains unknown.

Therefore, the purpose of this study was to characterize the current epidemiology, predominant injury mechanisms, associated injury patterns, delayed recognition, and in-hospital management of major trauma patients with THD. We hypothesized that the prevalence rate of THD remains low due to modern-day road and vehicle safety. Furthermore, we hypothesized a high prevalence of associated injuries and a low rate of missed THD due to a wide application of advanced imaging in contemporary trauma care.

## 2. Patients and Methods

### 2.1. TraumaRegister DGU^®^ and Data Acquisition

The TraumaRegister DGU^®^ (TR-DGU) of the German Trauma Society (Deutsche Gesellschaft für Unfallchirurgie) was founded in 1993 [16,17]. The aim of this multicenter database is to serve as a pseudonymized and standardized documentation of severely injured patients. Data are collected prospectively in four consecutive time phases from the site of the accident until discharge from the hospital: (A) prehospital, (B) emergency room and initial surgery, (C) intensive care unit (ICU), and (D) discharge.

The documentation includes detailed information on demographics, injury pattern, comorbidities, pre- and in-hospital management, the clinical course on ICU, relevant laboratory findings (including data on transfusion), and the outcome of each individual. The inclusion criterion is admission via the emergency room with subsequent intensive or intermediate care or death before admission to the ICU. The infrastructure for documentation, data management, and data analysis are provided by the Academy for Trauma Surgery (Akademie der Unfallchirurgie GmbH), a company affiliated with the German Trauma Society. Scientific leadership is provided by the Committee on Emergency Medicine, Intensive Care and Trauma Management of the German Trauma Society. The participating hospitals submit their pseudonymized data to a central database via a web-based application. Scientific data analysis is approved according to a peer review procedure established by Sektion NIS. The participating hospitals are primarily located in Germany (90%), but a rising number of hospitals from other countries contribute data as well (at the moment, Austria, Belgium, Finland, Luxemburg, Slovenia, Switzerland, the Netherlands, and the United Arab Emirates). Currently, approximately 30,000 cases from over 650 hospitals are entered into the database per year. Participation in the TR-DGU is voluntary. For hospitals associated with the TraumaNetzwerk DGU^®^, however, the entry of at least a basic dataset is obligatory for reasons of quality assurance. The present study is in line with the publication guidelines of the TR-DGU and is registered as TR-DGU project ID 2018-007.

### 2.2. Inclusion and Exclusion Criteria

**Figure 1:**
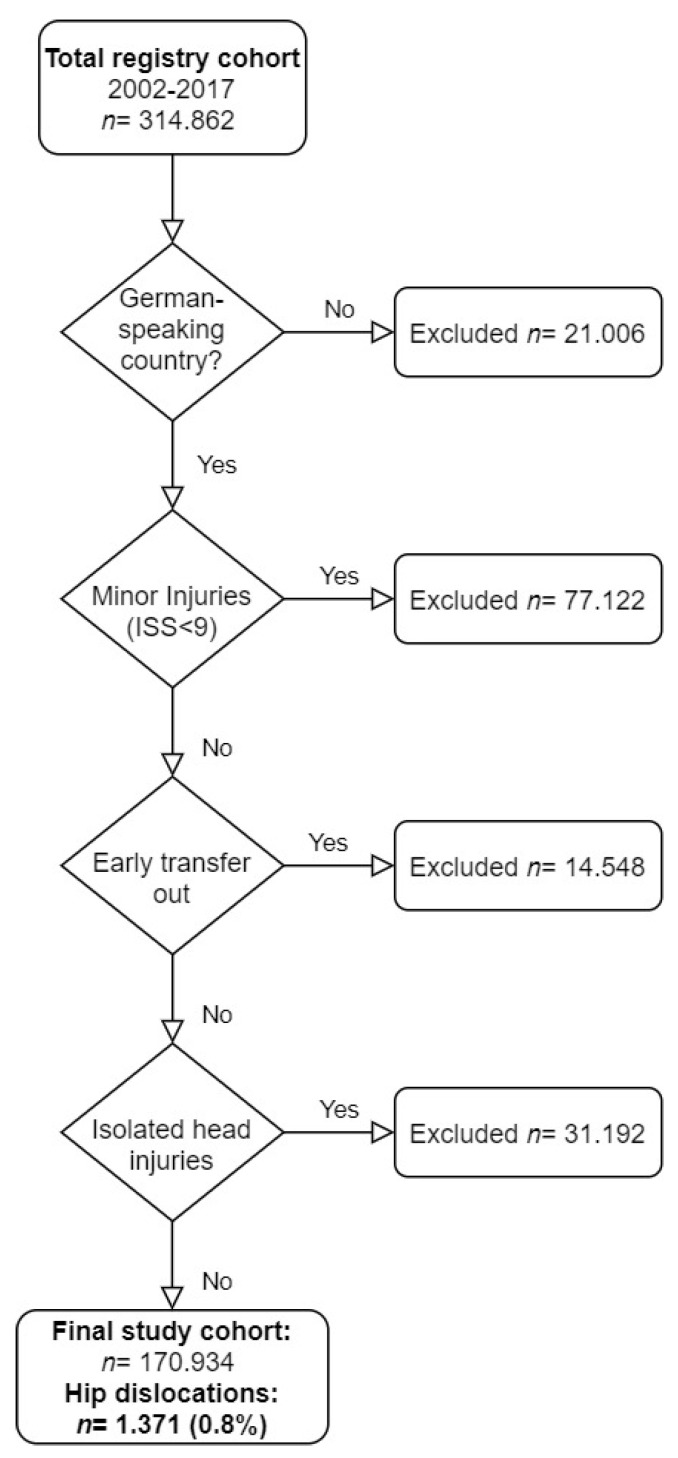
Description of the study cohort recruitment.

This analysis included data from trauma patients registered in the TR-DGU with major injuries (Injury Severity Score (ISS) ≥9 points) after admission to a participating trauma center in Germany, Austria, or Switzerland between January 2002 and December 2017 (Figure 1). Patients transferred “out” to another center within 48 h after admission were excluded due to missing outcome data and to exclude the risk of double counting from the receiving hospital. All cases transferred “in” were included to prevent bias in prevalence rates.

### 2.3. Definitions

#### 2.3.1. Mechanisms of Injury

According to the TR-DGU dataset, the following injury mechanisms were considered: (1) motor vehicle accident (MVA), (2) motorcycle accident (MCA), (3) bicycle accident, (4) pedestrian struck by a vehicle, (5) high fall (≥3 m), and (6) low fall (<3 m); further (combined) categories include (7) suicide attempt, (8) other, (9) blunt/penetrating trauma (not shown), and (10) traffic related (overall value presented).

#### 2.3.2. Injury Severity

According to the Abbreviated Injury Scale (AIS), the severity of injuries was documented as follows [18]: 1 (minor), 2 (moderate), 3 (severe, not life-threatening), 4 (serious, life-threatening), 5 (critical, survival uncertain), or 6 (maximum, currently untreatable). The ISS was derived from documented AIS values [19,20]. Since 2009, coding has followed a uniform protocol, and the data management processes has been previously described [21]. All injuries were coded according to the AIS Version 2005/Update 2008 (Association for the Advancement of Automotive Medicine, Barrington, IL, USA) [18,22].

#### 2.3.3. Identification of Traumatic Hip Dislocation and Ischial Nerve Compromise

Specific injuries and associated injuries were identified according to the AIS codes for THD (AIS 873030.2) and injuries of the ischial nerve (AIS 830499.2).

## 3. Statistical Analysis

Categorical data are presented as frequencies and percentages. Metric variables are reported as means and standard deviations (SDs). For skew distributed data, the median is also reported. Trauma patients without a THD served as a control group. Formal statistical testing was avoided due to the very large sample size. Logistic regression analysis was performed to evaluate the impact of various risk factors for THD. The results are presented as odds ratios (ORs) with 95% confidence intervals (CIs). The analysis was performed with SPSS (Version 25, IBM Inc., Armonk, NY, USA).

## 4. Results

### 4.1. Prevalence

**Figure 2:**
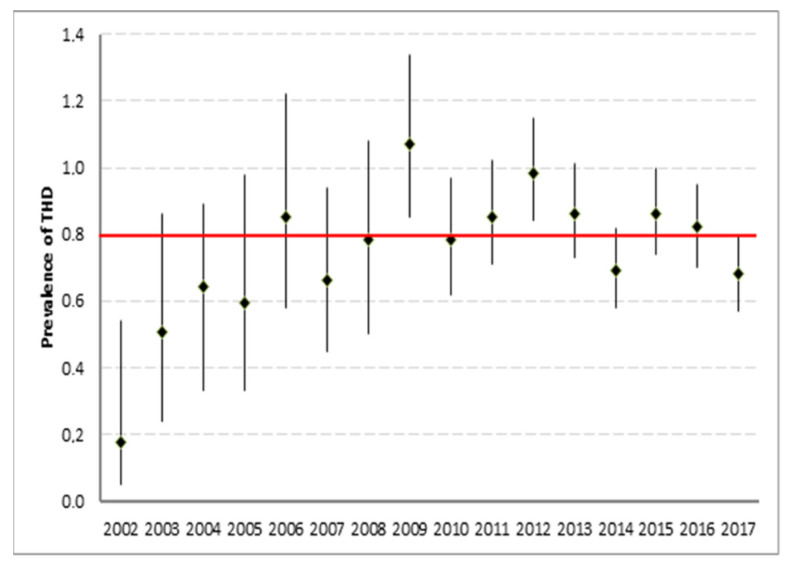
Annual prevalence of THD with 95% confidence interval.

According to the inclusion and exclusion criteria, a total of 170,934 trauma patients were included in the analysis (Figure 1). The proportion of patients who had sustained a THD was 0.8% (*n* = 1359). In 1359 patients with THD, a total of 1371 dislocated hips were observed; bilateral THDs were observed in 12 individuals. Between 4 and 176 THD cases were recorded per annum (Figure 2).

### 4.2. Demographic Data and Mechanisms of Injury

Major trauma patients with THD were most frequently injured in road traffic accidents (78.0%), especially in MVAs (49.9%). As compared with the control group, patients with THD were less frequently involved in bicycle accidents and fall mechanisms or struck as pedestrians (Table 1). Most patients with THD were male (79.5%, controls 71.5%) and had a mean age of 43 ± 19 years (controls 49 ± 22 years).

### 4.3. Injury Severity and Patterns

In patients with THD, a lower rate of head, facial, and spinal injuries was observed; however, patients with THD presented more frequently with abdominal, pelvic, and lower extremity injuries (Table 2). In particular, high rates of pelvic ring, acetabular, femoral, and patellar fractures were observed. A neurological deficit of the ischial nerve was found more frequently in patients with THD (*n* = 52, 3.8%) as compared with patients without THD (0.2%).

Overall, the THD group had a mean ISS of 22.4 points. The mean ISS and New ISS values of the control group were 0.7 points and 0.5 points lower, respectively, than the mean ISS and New ISS values of the THD group (Table 3).

Aortic injuries were observed more frequently in patients with THD (2.1%) as compared with the control group (0.9%). This difference was found to be statistically significant (*p* < 0.001).

### 4.4. Hospital Stay, Delayed Diagnosis, and Outcome Data

Despite a lower rate of head injuries and a comparable level of care measured by trauma center admission, the ICU stay and the total hospital stay were both prolonged in patients with THD. Most patients in both main groups required intensive care. A delayed diagnosis of a THD after ICU admission was documented in 1.1% of patients. Therefore, only a very small number of patients (*n* = 7) received a delayed diagnosis for THD.

Surgical management for THD was performed in 70.9% of patients. Patients with THD were often discharged fully recovered (48.2%) or experienced minor impairments (33.5%). The predicted (8.7%) and observed mortality (7.8%) were both lower in patients with THD as compared with the control group; however, average direct treatment costs were significantly increased.

### 4.5. Logistic Regression Analysis

The three injury mechanisms associated with the highest risk for THD (Table 4) were identified as: (1) MVAs (OR 2.98, 95% CI 2.57–3.45, *p* ≤ 0.001); (2) MCAs (OR 1.99, 95% CI 1.66–2.39, *p* ≤ 0.001); and (3) suicide attempts (OR 1.36, 95% CI 1.06–1.75, *p* = 0.016). The following concomitant injuries were observed in association with a present THD: (1) acetabular fracture (OR 10.85, 95% CI 9.21–12.76, *p* ≤ 0.001); (2) ischial nerve injury (OR 5.40, 95% CI 3.83–7.61, *p* ≤ 0.001); (3) pelvic fracture (OR 1.82, 95% CI 1.54–2.16, *p* ≤ 0.001); (4) femur fracture (OR 1.50, 95% CI 1.32–1.70, *p* ≤ 0.001); (5) patella fracture (OR 1.46, 95% CI 1.13–1.88, *p* = 0.003); and (6) aortic injury (OR 1.41, 95% CI 0.95–2.08, *p* = 0.090).

## 5. Discussion

### 5.1. Prevalence

In the present study, the overall annual prevalence of THD was 0.8% in major trauma patients. While there was an increase in the total number of THDs (4–176 cases/year) captured by the registry over time, the annual prevalence ranged between 0.6% and 1.1% in the most recent study period. The increasing prevalence in the first years of the study period may be related to a growing number of participating trauma centers in the multicenter database. After reaching the peak prevalence in 2009 (1.1%), the annual prevalence did not increase any further. Sahin et al. suggested an increasing incidence of this injury pattern in 2003 but referred to data published in *JAMA* in the early 1980s [5,23].

Regarding the injury mechanism, in 1979, Rosenthal et al. reported that 43/46 patients with posterior fracture-dislocations of the hip were unrestrained occupants in vehicular accidents and that 75% had sustained multiple injuries [24]. In 1999, Hak et al. observed that 55% of a study population of 36 patients were unrestrained and 95% had associated injuries mandating thorough evaluation [5]. Since multiple studies that have investigated the prevalence of THDs [1,2,5,11,25,26] were published before the current legislative regulations (e.g., mandatory seat belt use, and airbag deployment) and road safety initiatives were introduced, we sought to gather more actual epidemiological data. Based on the data of Haasper et al., who observed a low prevalence of “dashboard injuries” in only 5.8% of restrained car drivers in Germany (probably due to legislative measures) [27], it might be assumed that the number of THDs might have decreased over the last few years. However, we did not observe this development. Despite seat belt laws in effect, Cooper et al. suggested that most patients (57%) with hip dislocation evaluated between 2005 and 2015 were still unrestrained in Southern California [13]. In addition to this negative adherence to legislative measures, reciprocal effects of road traffic volume and density on the prevalence of THDs might also be assumed.

### 5.2. Delayed Diagnosis

Early diagnosis and prompt reduction in THDs are essential in the management of this true orthopedic emergency to prevent major complications and poor long-term outcomes [5,28,29]. Patients with THD typically present with acute pain, deformity with leg length discrepancy and/or malrotation, and the inability to bear weight. Therefore, a clear diagnosis is based on clinical and radiographic findings, and identification is only occasionally delayed. When the diagnosis of a hip dislocation is delayed for more than 72 h, the condition is defined as “neglected.” A Chinese study observed an average leg length discrepancy of 7.7 cm in 13 patients with neglected hip dislocation; therefore, a staged surgical protocol with an external fixation-assisted prereduction was performed [30]. The literature about both delayed and neglected THDs is scarce and mostly originates from the 1950s to the 1980s [31,32] or from low-income countries [31,33,34].

In the current study, most THDs were diagnosed early during the initial evaluation (98.9%), before admission to the ICU. Unconscious, pediatric, and hemodynamically unstable patients seem to be especially at risk for delayed or neglected diagnosis [32,35] and require special attention during the thorough evaluation in secondary and tertiary surveys as soon as life-threatening conditions are ruled out.

### 5.3. Demographic Data

Despite 40 years of road and vehicle safety evolution, patient characteristics appear to be highly comparable over time. Multiple studies have suggested that male patients were more frequently affected by traumatic dislocations of the hip [36]. As in our data, men were overrepresented (79.5%). However, demographic changes seem to affect the age pattern. Previous studies have reported a younger mean age in their series, ranging from 23.7 to 34.5 years [23,25,36,37]. The mean age of 43 years in the current study is significantly higher, which may indicate a changing demography of affected patients. A higher mean patient age of 37 years was also reported by a more recent study by Bhandari et al. (average 42 years), who evaluated 1076 cases from Dr. Matta’s registry, which captured patients who sustained acetabular fractures in the context of a posterior hip dislocation [7]. The available data from historical and current publications may suggest a trend toward an increasing patient age, as generally seen in trauma patients.

### 5.4. Mechanisms of Injury

Independent of the mechanism of injury, THD may result from an impact against the victim’s flexed knee, with the force transferred to the flexed hip along the femoral shaft. Higher degrees of hip flexion and adduction at the time of trauma typically result in simple dislocations, whereas less internal rotation or less adduction may result in fractures of the posterior acetabular wall [38]. Accordingly, a logistic regression analysis revealed independent associations between THD and pelvic (OR 1.82), femur (OR 1.50), and patellar fractures (OR 1.46) in our study. Historically, THDs have been frequently observed in the context of horse-riding activities. Today, traffic-related injury mechanisms, especially MVAs, constitute the leading cause of THD, as evidenced in this study (78%). In general, these findings are in line with the previous literature. Sahin et al. also reported traffic accidents as the major cause of THD (83.9%) in a small series of 52 cases [23].

In our data, the three mechanisms of injury with the highest risk for THD were identified as MVAs (OR 2.98, *p* ≤ 0.001), MCAs (OR 1.99, *p* ≤ 0.001), and suicide attempts (OR 1.36, *p* = 0.016). In a Taiwanese study of 96 fracture-dislocations of the hip, MCAs (*n* = 40) were the most common injury mechanism. This difference may be explained by a higher percentage of motorcycles in the regional traffic volume [39].

In addition to traffic accidents, which are well captured within the registry, recent reports have also described THDs in the context of winter sports activities, including sledging [40] and bicycle accidents [34]. A recent study observed that posterior hip dislocations may also occur in adolescents during sports activities in the absence of high-energy mechanisms, especially if acetabular retroversion and decreased posterior acetabular coverage were present [38].

In contrast to our results, previous studies have not yet reported an increased risk related to suicide attempts. It might be assumed that this association is predominantly caused by falls from great heights. However, in our study, both high- and low-level falls were more common in the control group when compared to the THD group.

### 5.5. Injury Severity and Patterns and Concomitant Injuries

Suraci et al. evaluated 38 patients with THDs in the 1980s and reported a mean ISS of 22.3 (SD 12.4), which is quite similar to the injury severity in our series (ISS 22.4, SD 12). Various studies have confirmed a high rate of multiple injuries [36,39], suggesting that THD must be considered to be an “indicator injury” for high-energy trauma. It also seems worth noting that, in our study, the abdomen, pelvis, and extremities were more frequently affected in patients with THD as compared with the control group. Therefore, special attention has to be paid to the presence of THD in cases of these injury patterns.

With a further focus on concomitant injuries, Hak et al. reported musculoskeletal (33%), head (24%), facial (21%), thoracic (21%), and abdominal trauma (15%), as well as femur fractures (14%), as the most frequent injury patterns [12] as compared with our results, in which we observed a higher rate of head (31.7%), chest (64.4%), and abdominal (26.7%) injuries. Potentially, a more liberal application of computed tomography with more detailed diagnosis of injuries in the current study period contributed to these findings.

We observed a combination of THD with an acetabular fracture in 48.7% (*n* = 667) of patients, whereas acetabular fractures were less frequently observed in the control group (4.4%, *n* = 7407). Furthermore, we found an independent association between THD and acetabular fractures (OR 10.85) in the logistic regression analysis. Cooper et al. found a comparable rate of combined acetabular fractures (53.92%) [13]. However, other studies have reported higher rates of an associated acetabular fracture in THD cases [41]. In this context, up to 70% of THDs were associated with an acetabular fracture.

In the current study, the presence of a THD was significantly associated with the risk for aortic injuries (OR 1.41). A study by Cooper et al. involved special attention on combined aortic injuries and THD, but this injury pattern was diagnosed in only one individual (0.8%). Therefore, further analysis was impossible [13]. When comparing the current and previous patient series with this combined injury pattern, significant differences regarding the mean injury severity are striking. Marymont et al. reported about seven patients with combined aortic injury and THD, with a mean ISS of 29 points (range of 25–45), while the series reported by Cooper et al. and Hak et al., both involving a single patient with aortic injury, had a mean ISS of only 13.49 points and 17.4 points (range of 9–59), respectively [12,13,15]. In the current study, the mean ISS for patients with THD was 22.4 points and, therefore, is more comparable to the Marymont et al. and Suraci et al. cohorts [15,25]. Different patient characteristics may explain the significant variances in the reported prevalence rates for aortic injuries between 0.8% and 8%. Aortic injuries are a common cause of early death after blunt trauma, and only 15–20% of patients with traumatic disruptions of the aorta survive the initial injury. The incidence of early mortality within 24 h is reported to be 30% [24]. Traumatic aortic injuries are a rare injury pattern, in consequence, statistical analysis remains challenging and non-significant *p*-values may be calculated in regression analysis. In addition to the mean injury severity, the quality and structure of prehospital care is also an important factor. Unfortunately, early mortality and the prehospital setting are not covered by most studies. The true prevalence of this combined injury pattern remains unknown and may underlie regional variances. However, aortic injuries are a very time-sensitive injury pattern and must not be missed. The trauma team should maintain a high index of suspicion for associated injuries in patients with THD to facilitate early detection and prompt management.

In our series, an ischial nerve injury was registered in 3.8% (*n* = 52) of patients. Furthermore, THD was significantly associated with the risk for ischial nerve injuries (OR 5.40). Potentially, this value obtained from the registry is still underreporting the real nerve injury rate. Cornwall and Radomisli reviewed the literature and suggested a higher incidence after THD and fracture-dislocation of the hip, approximately 10% in adults and 5% in children [6]. Fassler et al. precisely described sciatic nerve injuries associated with acetabular fractures, especially if the femoral head was dislocated posteriorly [26]. The authors observed that the peroneal division of the sciatic nerve was clinically involved in every patient with posterior THD. Furthermore, after electromyography analysis, the group suggested that both traumatic and iatrogenic nerve injuries were due to axonotmesis. In the past literature, a wide range of both sensory and motor symptoms have been described, arising either initially posttraumatic, perioperative (iatrogenic), or even postoperative as a result of complications (e.g., heterotopic ossifications and scaring) [9,42].

The presence of a THD increased the hospital length of stay and direct treatment costs. In the light of comparable injury severity, impaired mobility and weight-bearing restrictions associated with hip dislocations may also contribute to an extended hospital length of stay. Increased indirect costs have to be expected as a result of major long-term complications and higher rehabilitation needs in the context of a higher degree of impairment after hospital discharge. To the best of our knowledge, this significant socioeconomic impact of THD has not been specifically analyzed.

### 5.6. Limitations and Strengths

The current study has several strengths to be recognized. First, to the best of our knowledge, this is the largest study analyzing THDs in patients with major injuries. Therefore, the epidemiologic data, description of injury mechanisms, general injury severity, and concomitant injuries seem to be valid and clinically relevant. Second, the logistic regression analysis involved a wide range of risk factors, including injury mechanisms and associated injuries. The study characterizes the main variables associated with poor outcomes, including the rate of fracture-dislocations, delayed recognition, neurological deficits, and concomitant injuries. Furthermore, a trend toward an increasing patient age can be derived from our data and the historical literature. This finding may result in increased complication rates, arthroplasty procedures, and treatment costs.

We have to acknowledge the limitations of the present study. Due to the inclusion criteria of the TR-DGU, high-energy injuries were especially captured. The role of low-energy hip dislocations cannot be adequately assessed by the database. However, in accordance with the available literature, we consider low-energy hip dislocations in the context of anatomical variations as a rare entity. Unfortunately, the direction of hip dislocation and seat belt use are not captured within the registry. However, it is well known that >90% of THDs occur posteriorly and that unrestrained road traffic victims are overrepresented within victims of THDs. Furthermore, associated intraarticular (e.g., labral, osteochondral, and ligamentum teres) injuries, the time until closed reduction, and detailed functional outcome parameters are not documented within the registry. In the past years, these concomitant periarticular injuries (e.g., labral tears, osteochondral injuries, and loose bodies) have especially attracted further attention, as they may be addressed by arthroscopic techniques [37,43,44,45]. However, the current data suggest that special considerations are required in many patients with associated pelvic ring or lower extremity fractures to avoid complications associated with traction, patient positioning, and fluid extravasation [46,47]. Late complications after THDs have been characterized comprehensively within the literature. In particular, missed injuries, aortic injury, and neurological deficits may be underreported in multiply injured patients due to early mortality.

Sciatic nerve injuries may occur as a result of traumatic injury or as iatrogenic injury related to surgical procedures. The rate of sciatic nerve injuries in this study appears to be low; however, we are able to present a very large number of patients with THDs. A possible overestimation of temporary/transient nerve deficits in previous studies must also be considered. We suppose that complete or persistent ischial nerve palsies have especially been captured within the registry, or injuries were not diagnosed due to associated injuries or due to the high prevalence of head and spinal injuries in the THD group.

## 6. Conclusions

THDs are frequently related to high-energy mechanisms and associated with severe concomitant injuries in major trauma patients. THDs are occasionally missed during initial evaluation. However, this injury pattern requires surgical management in most cases and represents a challenging condition, especially in multiply injured individuals.

## Figures and Tables

**Table 1:** Mechanisms of Injury.

	Control Group *n* = 166,420	Hip Dislocation *n* = 1349	Total
Motor vehicle accident % (*n*)	25.1% (41,702)	49.9% (673)	25.3% (42,375)
Motorcycle accident % (*n*)	15.2% (25,283)	18.0% (243)	15,2% (25,526)
Bicycle accident % (*n*)	8.3% (13,883)	2.9% (39)	8.3% (13,922)
Pedestrian struck % (*n*)	7.4% (12,251)	5.6% (75)	7.3% (12,326)
High fall ≥3m % (*n*)	17.7% (29,392)	11.5% (155)	17.6% (29,547)
Low fall <3m % (*n*)	16.1% (26,784)	4.2% (56)	16.0% (26,840)
Traffic-related (all) % (*n*)	57.2% (95,137)	78.0% (1349)	57.3% (96,189)
Suicide attempt % (*n*)	4.9% (8142)	6.0% (80)	4.9% (8222)
Other % (*n*)	10.3% (17,125)	8.0% (108)	10.3% (17,233)

**Table 2:** Patterns of injury.

	Control Group	Hip Dislocation	Total
Head injury (AIS ≥ 2)	42.3% (71,669)	31.7% (435)	42.2% (72,104)
Facial injury (AIS ≥ 2)	14.3% (24,215)	11.4% (156)	14.3% (24,371)
Neck injury (AIS ≥ 2)	1.2% (2108)	1.2% (16)	1.2% (2124)
Chest injury (AIS ≥ 2)	61.9% (104,898)	64.6% (886)	61.9% (105,784)
Abdominal injury (AIS ≥ 2)	20.5% (34,720)	26.8% (368)	20.5% (35,088)
Spinal injury (AIS ≥ 2)	34.0% (57,688)	30.3% (415)	34.0% (58,103)
Extremity injury (AIS ≥ 3)	34.7% (58,804)	53.9% (739)	34.8% (59,543)
UE injury (AIS ≥ 2)	37.5% (63,638)	35.2% (482)	37.5% (64,120)
LE injury (AIS ≥ 2)	32.9% (55,866)	100% (1371)	33.5% (57,237)
Pelvic injury (AIS ≥ 2)	21.1% (35,828)	63.3% (872)	21.5% (36,700)
Acetabular fracture % (*n*)	4.4% (7407)	48.7% (667)	4.7% (8074)
Femur fracture % (*n*)	16.4% (27,783)	30.5% (418)	16.5% (28,201)
Patella fracture % (*n*)	1.9% (3217)	5.8% (80)	1.9% (3297)
Ischial nerve injury % (*n*)	0.2% (280)	3.8% (52)	0.2% (332)
Aortic injury % (*n*)	0.9% (1588)	2.1% (29)	0.9% (1617)

UE, upper extremity; LE, lower extremity.

**Table 3:** Demographic, treatment, and outcome data.

	Control Group	Hip Dislocation
Male sex % (*n*)	71.5% (121,053)	79.5% (1087)
Mean age (SD), years	49 (22)	43 (19)
ISS: mean (SD), points	21.7 (12)	22.4 (12)
New ISS: mean (SD), points	26.2 (14)	26.7 (13)
Level 1 center admission % (*n*)	66.8% (113,218)	66.5% (912)
Level 2 center admission % (*n*)	24.9% (42,289)	26.6% (365)
Level 3 center admission % (*n*)	8.3% (14,056)	6.9% (94)
ICU treatment % (*n*)	88.2% (149,471)	93.1% (1277)
ICU LOS days †	7.5/3 (11)	9.1/4 (13)
Ventilator, mean days (SD)	3.7 (9)	4.6 (9)
Hospital LOS: days †	20/14 (21)	27/22 (24)
Delayed identification β	NA	1.1% (7)
Surgery for dislocated hip % (*n*)	NA	70.9% (518)
Discharged fully recovered % (*n*)	56.3% (92,186)	48.2% (639)
Discharged with minor impairment % (*n*)	23.6% (38,560)	33.5% (444)
Discharged with severe impairment % (*n*)	7.7% (12,540)	9.0% (119)
Discharged with PVS	1.4% (2229)	1.2% (16)
Predicted mortality (RISC II)	10.8%	8.7%
Observed mortality % (*n*)	10.7% (18,216)	7.8% (107)
Mean treatment costs (Euro)	18,600	24,659

LOS = length of stay; ICU = intensive care unit; NA = not applicable; HD = Hip dislocation, RISC II = Revised Injury Severity Score II; PVS = persistent vegetative state; (†) mean/median (SD); (β) Hip dislocation diagnosed at ICU or later; available for cases with standard documentation only.

**Table 4:** Logistic regression analysis with hip dislocation as dependent variable: Injury mechanism and concomitant injuries.

Risk Factors	Odds Ratio (OR)	95% Confidence Interval (CI)	*p*-Value
Injury mechanism			
Motor vehicle accident	2.98	2.57–3.45	<0.001
Motorcycle accident	1.99	1.66–2.39	<0.001
Pedestrian struck	1.22	0.94–1.59	0.130
Bicycle accident	0.80	0.57–1.12	0.184
Suicide attempt	1.36	1.06–1.75	0.016
Concomitant injuries			
Acetabular fracture	10.85	9.21–12.76	<0.001
Ischial nerve injury	5.40	3.83–7.61	<0.001
Pelvic fracture	1.82	1.54–2.16	<0.001
Femur fracture	1.50	1.32–1.70	<0.001
Patella fracture	1.46	1.13–1.88	0.003
Injury of the aorta	1.41	0.95–2.08	0.090

## Data Availability

The data that support the findings of this study are available from the Academy for Trauma Surgery (Akademie der Unfallchirurgie GmbH), a company affiliated with the German Trauma Society, but restrictions apply to the availability of these data, which were used under license for the current study according to the publication guidelines of TraumaRegister DGU^®^ and so are not publicly available. Data are, however, available from the authors upon reasonable request and with the permission of the Academy for Trauma Surgery and the TraumaRegister DGU^®^ Review Board.
